# An improved approach for automated cervical cell segmentation with PointRend

**DOI:** 10.1038/s41598-024-64583-7

**Published:** 2024-06-20

**Authors:** Baocan Zhang, Wenfeng Wang, Wei Zhao, Xiaolu Jiang, Lalit Mohan Patnaik

**Affiliations:** 1 Chengyi College, Jimei University, Xiamen, 361021 Fujian China; 2 Shanghai Institute of Technology, Shanghai, 200235 China; 3 London Institute of Technology, International Academy of Visual Art and Engineering, London, CR2 6EQ UK; 4 National Institute of Advanced Studies, Bangalore, 560015 India

**Keywords:** Cytoplasm segmentation, PointRend, Overlapping cervical cells, Mask RCNN, ISBI, Computational biology and bioinformatics, Health care, Mathematics and computing

## Abstract

Regular screening for cervical cancer is one of the best tools to reduce cancer incidence. Automated cell segmentation in screening is an essential task because it can present better understanding of the characteristics of cervical cells. The main challenge of cell cytoplasm segmentation is that many boundaries in cell clumps are extremely difficult to be identified. This paper proposes a new convolutional neural network based on Mask RCNN and PointRend module, to segment overlapping cervical cells. The PointRend head concatenates fine grained features and coarse features extracted from different feature maps to fine-tune the candidate boundary pixels of cell cytoplasm, which are crucial for precise cell segmentation. The proposed model achieves a 0.97 DSC (Dice Similarity Coefficient), 0.96 TPRp (Pixelwise True Positive Rate), 0.007 FPRp (Pixelwise False Positive Rate) and 0.006 FNRo (Object False Negative Rate) on dataset from ISBI2014. Specially, the proposed method outperforms state-of-the-art result by about $3\%$ on DSC, $1\%$ on TPRp and $1.4\%$ on FNRo respectively. The performance metrics of our model on dataset from ISBI2015 are slight better than the average value of other approaches. Those results indicate that the proposed method could be effective in cytological analysis and then help experts correctly discover cervical cell lesions

## Introduction

Globally, cervical cancer is the fourth leading cause of death in women^1^. Cervical cytopathology tests can detect the cervical cancer in its early stage, which is essential for successful treatment. Cervical cancer screening techniques include papanicolaou test (Pap), human papillomavirus (HPV) test and histopathology test. New manual screening techniques such as TCT (Thinprep Cytologic Test) have been widely used nowadays to detect cancer. Traditionally, human experts examine the slides carefully under microscope, to determine the degree of pre-cancerous lesions^2^. But this process is likely to be affected by many factors such as fatigue of eyes, resulting in misdiagnosis. So more robust automatic cervix type classification^3^ and cervical cell segmentation techniques are much needed. Cell segmentation can present much more detailed information about cervical cells than cervix type classification, to make it one of the most important techniques in cervical cancer screening.

The detection of morphological features of the nucleus and cytoplasm is the core task of cervical cell pathological examination. Cytoplasmic characteristics have been shown to be critical for identifying abnormal cells^4^. The precise segmentation of cytoplasm in cervical cytology images is the essential part of cell segmentation. Once the edge of cytoplasm is located, quantitative evaluation such as cell diameter and rate of cell deformation can be calculated. Based on this information, pathologists can determine the degree of cervical cell lesions. Especially accurate segmentation of cytoplasm from cell clumps of high density in real EDF images can clearly present the shape and volume characteristics of cervical cells, which could tremendously improve the effectiveness of examining cervical cell screening clinically.

In recent years, computer-aided diagnosis system has been utilized by pathologists to speed up the process of screening and examining. Generally, actual cervical cytology images often contain mucus, blood or other debris, and the cells in the images sometimes overlap with each other at a very high rate. In some cases, cell boundaries in clumps are extremely difficult to be identified, or even visually indistinguishable. Those facts make it a challenging task to precisely segment every single cell in the cervical cell images. Meanwhile, cell segmentation plays a very important role in detecting the early stage of cervical cancer. Thus, automated cervical cell segmentation has became an essential area of interest. Many scholars around the world have proposed various methods for cell instance segmentation, most of which are based on deep learning^5–8^. In general, deep learning models need a large number of samples and corresponding annotations for training. However, manual annotations of high quality are very tedious and time consuming. Thus biomedical datasets are very rare and precious. The IEEE international Biomedical Imaging Society held the first and second cervical cell image segmentation challenges in 2014 and 2015, and published two high quality datasets containing cervical cytology images and manual annotations. Those two datasets have motivated the study of cervical cell segmentation greatly. For example, the widely cited method proposed by Tareef^9^ is evaluated on those two datasets.

However, the two high quality datasets published on ISBI2014 and ISIB2015 only have 962 images in total. They are not enough for modern networks. So transfer learning is usually used in the training process, to overcome the scarcity of samples. Namely, many models are initialized by weights pre-trained on large scale datasets (such as ImageNet, COCO datasets^10^). Also other data augmentation techniques such as affine transformation and image cropping are utilized in the training process of neural networks.

This research introduces a new deep learning model based on Mask RCNN and PointRend module for cell segmentation, aiming to improve the performance of segmenting. The main contributions of our work are:

- We propose a method to segment cytoplasm from cell clumps in cervical cytology images. The model is created by adding a PointRend module to the traditional Mask RCNN. This new branch uses fine grained features and coarse features extracted from different feature maps to fine-tune the boundary pixels of low certainty. To our knowledge, this work is the first to deal with cell segmentation using PointRend module.
- Meticulous experiments have been carried out to find the best architecture. As a result, our model outperforms state-of-the-art approaches on four widely adopted metrics.

The rest of this paper is organized as follows. Section "Related works" briefly discusses previous works about cell segmentation; Section "Method" gives the structure of the proposed convolutional neural network; Section "Results" presents the detailed information of datasets, the main results achieved and comparison with other approaches; The discussion is presented in "Discussion"; Finally, the conclusion of our study and possible methods to improve segmentation performance are given in "Conclusion and future works".

## Related works

As for regular screening for cervical cancers, previous works often focus on nuclei segmentation, cytoplasm segmentation and cervix type classification on datasets of various images (such as Pap images, colposcope images). Cervical cell segmentation is a very challenging task even for human experts. But it’s essential to obtain good enough performance for cytological problems, in order to assist in real diagnostic process. Many previous researches in cervical cell segmentation use classical image analysis methods. Zhang et al.^11^ proposed a scheme based on graph cut to segment cervical cells and achieved 0.93 accuracy for cytoplasm. Win et al.^12^ combined several algorithms such as watershed and random forests, to segment nuclei and cytoplasm. They tested the method on Herlev dataset, and achieved 0.86 on the metric DSC. Rasheed et al.^13^ proposed a deep learning model named Cervical-Net to segment cervical nuclei from overlapped cervical cell smear images. They used a bi-directional feature pyramid network to learn spatial and local features, in order to strengthen the traditional UNet. They achieved impressive results with Dice coefficient of 0.93, pixel-level accuracy of 0.93, and object-level recall of 0.95. On the other hand, deep learning based models have been widely used in the task of cervical cell classification. Habtemariam et al.^3^ used a lightweight MobileNetv2-YOLOv3 model to detect the transformation region. Then the extracted features were fed into the EffecientNetB0 for cervix type classification. They used both histopathology images and colposcope images for classification in their work. Zewde et al.^14^ used traditional convolutional neural network ResNet152 to classify pap smear images into histopathology-based cervical cancer type. They also developed an online system to be used clinically. Another publicly available dataset SIPaKMed^15^, which consists of 4049 segmented pap smear images, is often used as evaluation dataset too (such as in Hemalatha’s work^16^).

After the two important datasets (ISBI2014 and ISBI2015) were made publicly available, most of the methods for segmenting cervical cells have been evaluated on those two datasets. Ushizima et al.^17^ obtained cell clumps and nucleus regions based on the similarity of adjacent pixels. It can only divide overlapping cells by straight lines. Phoulady et al.^18^ proposed a method based on iterative threshold and the regularization level set algorithm. They segmented the cell clumps and nucleus areas by iterative threshold, and then obtained the smooth cytoplasmic boundaries by the regular level set assumed by the ellipse shape. To further improve the performance of cytoplasm segmentation, Phoulady et al.^19,20^ detected new candidate boundary points by defining a weight vector, and used a smoothing filter to smooth the candidate boundary points. The main contribution of this work was that the depth information of the stacked cell images was used to obtain a more precise cytoplasm boundary. Wang et al.^21^ proposed a tree domain structure and screening algorithm based on depth-first searching strategy to obtain the candidate masks of nuclei. Song et al.^22^ constructed the segmentation by grouping contour fragments to form a closed boundary, where shape priors such as curvature information were used. Wang et al.^23^ proposed a segmentation algorithm based on the nuclear radial boundary enhancement for overlapping cells. Although deep learning based methods generally are computationally expensive, they outperform other algorithms in many computer vision tasks. As for cervical cytoplasm segmentation, some methods based on convolutional neural network have been proposed. Song et al.^5^ presented a multi-scale CNN to classify every pixel into cytoplasm or background, where the accurate detection of nuclei was critical. They also incorporated high-level shape information to guide segmentation. Tareef et al.^6^ presented a segmentation framework on super-pixelwise convolutional neural network and utilized a learning shape prior to delineate the contour of each individual cytoplasm mask. Wan et al.^7^ adopted TernausNet to classify the image pixels into nucleus, cytoplasm or background. Then Wan proposed a modified DeepLab model to perform cytoplasm segmentation. Hao et al.^8^ constructed a CRP-PSN deep learning network, where CRP aimed to reduce background noise and impurities. Our previous work^24^ introduced a new model based on Mask RCNN to segment cytoplasm from cell clumps.

## Method

### Datasets

In 2014 and 2015, the first and second Overlapping Cervical Cytology Image Segmentation challenges (ISBI 2014 and ISBI 2015^25^) were hold, with two high-quality datasets of cervical cytology images and their ground-truth segmentation made publicly available. Those two challenges greatly have motivated the research of overlapping cell segmentation. The first dataset (from ISBI 2014) consists of 135 synthetic (45 images for training and 90 images for validating) and 8 real cervical cytology extended depths of field (abbreviated as EDF) images in the training set and 810 synthetic and 8 real cervical cytology EDF images in the testing set. The synthetic images are created by minor transformation of background and brightness of different annotated isolate cells in real EDF images. It’s should be noticed that the real EDF images in this dataset were released without annotations of individual cytoplasm. So those images were not used in our paper. The second dataset (from ISBI 2015) contains 8 real cervical cytology EDF images in the training set and 9 real ones in the testing set, with both ground-truth annotations of cytoplasm.

As for the main difference of those two datasets, images from ISBI 2014 have size $512\times 512$ with 2–10 cells in each image, and images from ISBI 2015 have size $1024\times 1024$ with more than 40 highly overlapped cells in each image. Table 1 presents the partitioning of datasets used in our paper. Concretely, the training dataset used in this paper consists of 855 synthetic cell images from ISBI2014 and 8 EDF images from the training set of ISBI2015. The testing dataset is composed of 90 synthetic images for validating from ISBI2014 and 9 EDF images from testing set of ISBI2015.

**Table 1:** The number of cell images in two datasets and their partitioning.

	Total	ISBI2014	ISBI2015
Training set	863	855	8
Testing set	99	90	9

Data augmentation can improve generalization ability of deep learning model, and also can reduce probability of overfitting. Geometric transformations (rotation, vertical and horizontal flipping, and scaling) were applied in the training process, where each of the transformations was selected and applied randomly.

**Figure 1:**
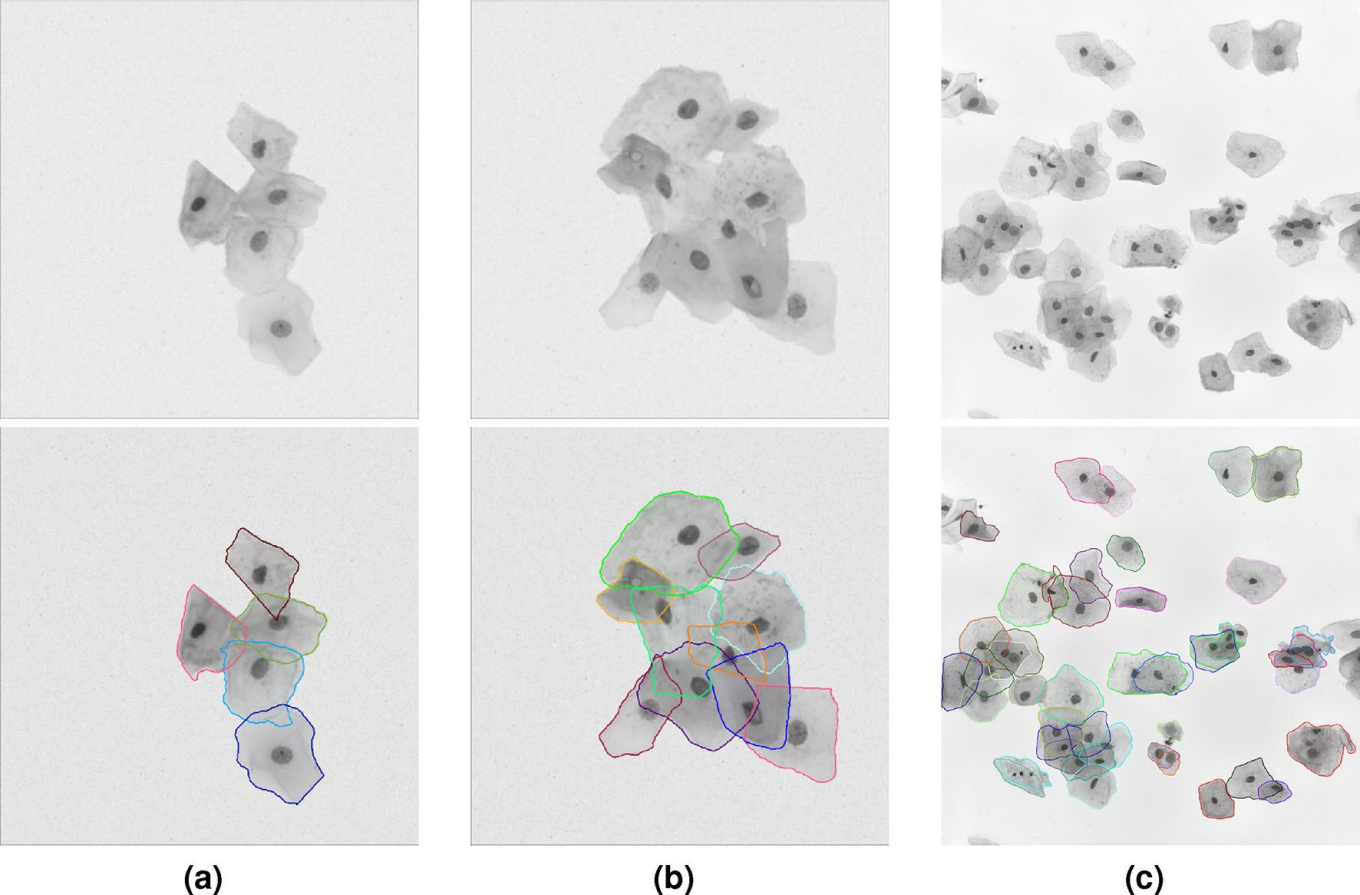
Typical cytology images (first row) with their ground-truth masks (second row) in the training dataset: (**a**) synthetic cell image with low overlap rate (from ISBI2014); (**b**) synthetic cell image with moderate overlap rate (from ISBI2014); (**c**) real EDF cell image with high overlap rate (from ISBI2015).

Samples of cytology images and their masks are presented in Fig. 1. These images show challenges those datasets present for cell segmentation. As can be seen in the real EDF image, multiple cells overlap at a relatively high rate, which makes precisely segmenting cells from the clumps very hard. Even it’s a very challenging task for human expert to correctly label all cell boundaries. Finally, masks could have defects, such as non-labeled pixels.

### Structure of the model

There are two major categories of methods for instance segmentation, one stage methods such as yolo5 and two stage methods such as Mask RCNN. In recent years, instance segmentation methods based on Mask RCNN meta-architecture^26^ have been proven to be very effective. These region-based deep learning models typically predict masks of size $28\times 28$, irrespective of the sizes of input images. This is sufficient for the coarse detection of moderate objects, as the cell cytoplasm in cervical cell images. However, the edge of cell instance is harder to predict than interior pixels (nuclei), and is more likely to be inferred wrongly. In this paper, we focus on the improvement of edge detection accuracy in cervical cell segmentation.

**Figure 2:**
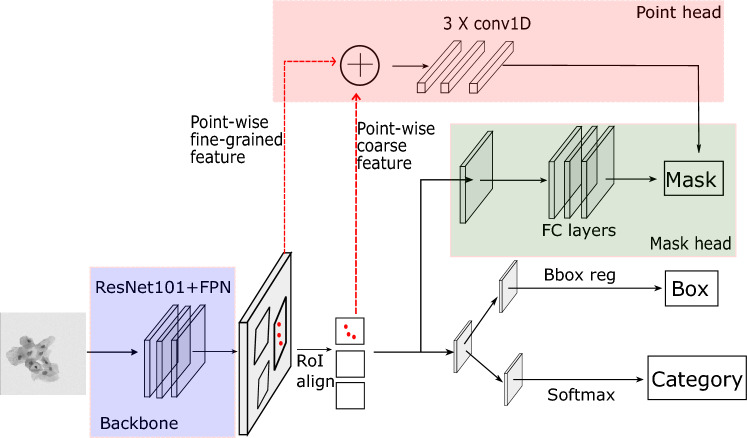
The architecture of neural network with PointRend module. The red points in proposals and coarse features (the outputs of RoI align) are the selected points. Two point-wise feature maps are extracted and concatenated.

We propose a new module based on PointRend^27^ for improving edge detection accuracy, and then use this module with Mask RCNN to segment cell cytoplasm in cervical cytology images. The architecture of the model is depicted in Fig. 2. In detail, the traditional two stage instance segmentation model Mask RCNN consists of three branches, category branch, box regression branch and mask branch. The backbone network is ResNet101 with Feature Pyramid Network (FPN), with weights pre-trained on COCO datasets. Let

$$P_2, P_3, P_4, P_5 =\mathcal {F}(X)$$

where *X* is the array of input image and $\mathcal {F}$ is the function of backbone network with FPN, and $P_2, P_3, P_4, P_5$ are the exacted feature maps. For each candidate anchor, the feature (proposal) is obtained from corresponding feature map (one of $P_2, P_3, P_4, P_5$) and then refined by the region proposal network (RPN). For each proposal, a feature of size $7\times 7$ is obtained by RoI align, instead of RoI pooling. The value of a pixel in the $7\times 7$ feature is calculated by bilinear interpolation, whose definition is as followed.

$$\begin{aligned} \varPhi (x,y) = (x-x_1)(y-y_1)\varPhi (x_2, y_2) +&(x-x_2)(y-y_1)\varPhi (x_1, y_2) +&(x-x_1)(y-y_2)\varPhi (x_2, y_1) + (x-x_2)(y-y_2)\varPhi (x_1, y_1) \end{aligned}$$

where $(x_1, y_1), (x_1, y_2), (x_2, y_1), (x_2, y_2)$ are the four nearest pixels of (*x*, *y*) and $\varPhi$ means the value of a pixel. In the mask branch of Mask RCNN, the feature maps $X'$ of size $7\times 7$ are interpolated to get output $Y'$ of size $28\times 28$. Upsampling is implemented by applying transposed convolution iteratively:

$$\begin{aligned}Y'= C^TX'\end{aligned}$$

where *C* is the convolution kernel. Because the mask of size $28\times 28$ may not be sufficient for accurate detection of cell cytoplasm, a new module is used with the mask branch to fine-tune the predictions of masks. This new module (red rectangle area in Fig. 2) consists of three main components: (1) select a small number of real-value points of low certainty (a pixel of low certainty probably lies on the edge) to make predictions on by some strategy; (2) for each selected point, a point-wise feature is obtained by concatenating point-wise features from two feature maps. One is the feature map of size $7\times 7$ after RoI align module of the model Mask RCNN (coarse feature map). The other is the feature map produced by RPN module (fine-grained feature map); (3) Point head: a small neural network with three one-dimensional convolution layers to predict the labels of the selected points.

In detail, the red dots in Fig. 2 are the sampled points of low certainty. A point-wise feature, referred as fine-grained feature $F_{fine}$, is extracted from the feature maps generated by FPN. Because a point is a real-value coordinate, bilinear interpolation is used to compute the feature vector. The fine-grained feature $F_{fine}$ focuses on depicting the details of object, but dose not contain region-specific characteristics. As for the cell segmentation in cervical cytology images, cells overlap with a relatively high rate. A point may be labeled as foreground by one cell instance, but background by another cell instance. Different regions predict different labels for the same point. So region-specific features are needed. Therefor, a point-wise feature, referred as coarse feature $F_{coarse}$, is computed from feature maps generated by RoI align module, where the channels convey regional information. Then, a small neural network is applied on the concatenated feature maps, to get the output $Y''$ of point head. The small neural network consists of three one-dimensional convolution layers, each followed by a ReLu function. Namely,

$$Y''= ReConv1D(ReConv1D(ReConv1D(F_{fine}\oplus F_{coarse})))$$

where *ReConv*1*D* is the one-dimensional convolution function followed by a ReLu function. The output $Y''$ of point head is used to refine the boundary of binary mask predicted by mask branch. The total loss of our model consists of four parts: $L = L_{mask}+L_{point}+L_{box}+L_{cls}$, where $L_{mask}$, $L_{box}$, $L_{cls}$ is the loss of mask branch, box regression branch, and category branch respectively. $L_{point}$ is the binary cross entropy loss of point head, defined as:

$$\begin{aligned}L_{point}=-[y_n\log \sigma (x_n) + (1-y_n)\log (1-\sigma (x_n))] \end{aligned}$$

where $y_n$ equals 0 (background) or 1 (cytoplasm), $\sigma$ is the sigmoid function and $x_n$ is the predicted value.

Following statement further illustrates the impact of PointRend module. Precise detection of boundary pixels of cervical cell cytoplasm is crucial for pathologists to determine the degree of pre-cancerous lesions. Under this consideration, the PointRend module is cooperated into base model Mask RCNN, to improve the accuracy of edge detection. Fine-grained features from backbone network focus on general local characteristics and coarse features contain more contextual and semantic information. Then by concatenating those two features, a small network point head (PointRend module) is added to exclusively learn the feature of boundary pixels. In this way, the point head tends not to be affected by internal or background pixels. Finally, the output of point head is used to fine-tune the edge of cell cytoplasm predicted by the base model Mask RCNN.

### Implementation details

Publicly available datasets of cervical cytology images with high quality annotations are very rare. The widely used ISBI datasets for cervical cell segmentation only contain 962 images in total. Based on the above facts, the weights pre-trained on COCO datasets^10^ are used to initialize the model. Then the model is further trained and fine-tuned on the ISBI datasets. The base model Mask RCNN from detectron2 (of facebook) is used in this paper. In the training process, 196 (e.g. $14\times 14$) pixels of low certainty are selected randomly. Then the features of those pixels are calculated. Specially the fine grained features are extracted from $P_2$ feature map of the FPN network. After concatenating two features, three one-dimension fully convolutional layers of size 256 in Point head are used to make predictions for the most uncertain points (e.g. those with probabilities closest to 0.5 for a binary mask). During inference, 784 (e.g. $28\times 28$) points of low certainty are selected randomly to fine tune the edge pixels of cell cytoplasm. As for general training details, the base learning rate is 0.002 with momentum 0.9. The model is trained for 500 epochs in total, while data augmentations such as horizontal flip are enabled. The cytology cell images from ISBI2015 of size $1024\times 1024$ are resized. The size of batch normalization is 2 images per batch. The training and inference platform is a desktop system with 2 GPUs (one Nvidia RTX2080Ti and one RTX 3090) and 128G memory running Debian 11.

### Training and performance measures

In order to compare our segmentation results with the ones proposed by other researchers on the same datasets, we adopt four widely used evaluation metrics. The dice similarity coefficient (abbreviated as DSC) of two regions A and B is defined as:

$$\textrm{DSC} = 2\times \frac{|A\cap B|}{|A| + |B|}$$

where operator |.| means the area of a region. A cell in the ground truth is considered to be successfully segmented if a segmentation predicted by the model has DSC above a specific threshold with it. The rate of cells in ground truth without a predicted segmentation having a DSC above a specific threshold with it is defined as object-based false negative rate (abbreviated as FNRo). In this paper, we adopt the following values of DSC threshold: $\{0.7, 0.8\}$ (0.7 was the officially recommended DSC threshold in the two challenges). At pixel level, measures are computed for each pair of ground-truth segmentation $gt_i$ and predicted detection $det_j$. True positive (TP) is the number of pixels in $gt_i \cap det_j$. False negative (FN) is the number of pixels in $gt_i - det_j$. False positive (FP) is the number of pixels in $det_j - gt_i$. True negative (TN) is the number of pixels in the complementary set of $gt_i \cup det_j$. True positive rate (abbreviated as TRPp) and false positive rate (abbreviated as FPRp) are also reported in our paper, whose definitions are as followed:

$$\begin{aligned}\textrm{TPRp} =\frac{TP}{TP+FN},~~~ \textrm{FPRp} =\frac{FP}{FP+TN}. \end{aligned}$$

Higher values of TPRp with lower values of FPRp mean better cell segmentation. It should be noticed that during evaluation a cell in the ground truth is not counted in the metrics of TPRp and FRPp if there is no predicted segmentation that has a DSC greater than the specific DSC threshold with it. The metrics of COCO standard segmentation AP^10^ are also reported, including AP, AP50 and AP75.

## Results

### Evaluation and comparison

**Figure 3:**
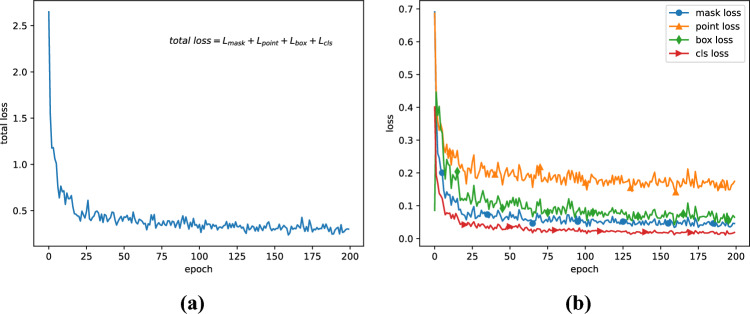
Experiment loss: (**a**) total loss performance; (**b**) four component losses.

Our purpose is to classify each pixel in cervical images into two classes: background or cytoplasm. The backbone network is ResNet101 with FPN, by using COCO weights for transfer learning. Loss performance per epoch is shown in Fig. 3.

**Figure 4:**
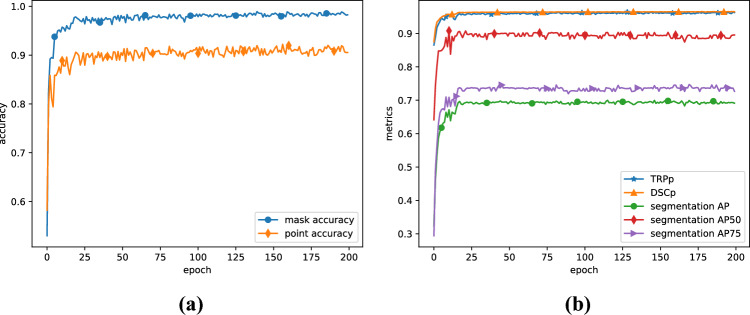
Experiment accuracy and metrics: (**a**) mask head accuracy and point head accuracy; (**b**) five metrics adopted in our experiment.

As can be seen from loss images, the model converges after about one hundred epochs, without any large fluctuations. It may imply that the model is well fitted. Figure 4 shows that the accuracy and metrics plateau early, causing the model to converge to a stable state quickly.

On the same datasets, many scholars have presented various overlapping cell segmentation methods, some of which achieved very impressive segmentation results. The detailed results of our model are presented in Table 2. The table aggregates all the results and computes the metrics by average. As for the whole testing dataset, the COCO standard segmentation AP, AP50 and AP75 are 0.685, 0.886 and 0.738 respectively.

**Table 2:** Segmentation performance with different DSC thresholds.

Dataset	Thresholding	DSC	TPRp	FPRp	FNRo
ISBI2014	DSC > 0.7	0.967 ± 0.024	0.965 ± 0.026	0.0007 ± 0.0006	0.006 ± 0.033
	DSC > 0.8	0.968 ± 0.021	0.965 ± 0.025	0.0007 ± 0.0005	0.013 ± 0.057
ISBI2015	DSC > 0.7	0.883 ± 0.021	0.903 ± 0.041	0.001 ± 0.0002	0.218 ± 0.123
	DSC > 0.8	0.917 ± 0.011	0.928 ± 0.026	0.0007 ± 0.0001	0.382 ± 0.149

The values are in the format of $\mu \pm \sigma$.

In the following, we focus on the analysis and comparison of various methods for cervical cytology segmentation. Our proposed method and other cell segmentation methods are compared quantitatively. The qualitative results of our model are also presented.

In Tables 3 and 4, not only classical results^9,25,28^ but also results published in recent years are included for comparison. As can be seen from Table 4, Tareef et al.^9^ achieved the highest value of TPRp but the rest metrics are moderate; Wan et al.^7^ presented a segmentation results with both DSC and TPRp above 0.9. The average of four metrics across the six compared results is DSC=0.88, TPRp=0.908, FPRp=0.0026 and FNRo=0.253. Our proposed results are all above the average. To the best of our knowledge, no published paper has achieved supreme results on all four metrics on the testing dataset from ISBI2015. On the other hand, Table 3 shows that our proposed method achieved the best results on all metrics on the dataset from ISIB2014. In detail, our proposed method outperforms the best results of previous works by 3% on DSC, 1% on TPRp and 1.4% on FNRo respectively. Our achieved FNRo is extremely low, which means our model predicts a mask for almost every cell with DSC above the threshold (0.7). Table 5 shows similar comparison results as Table 3, where the DSC threshold is 0.8. In summary, our proposed method outperforms state-of-the-art cervical cytology segmentation approaches on dataset from ISBI2014, and is a little bit better than the average segmentation performance of other methods on dataset from ISBI2015.

**Table 3:** Comparison of segmentation performance on ISBI 2014 testing dataset using DSC, TPRp, FPRp, FNRo(DSC threshold = 0.7).

Methods	DSC	TPRp	FPRp	FNRo
Tareef^9^	0.90 ± NA	0.95 ± NA	0.004 ± NA	0.11 ± NA
Lu^25^	0.94 ± 0.07	0.95 ± 0.07	0.004 ± 0.01	0.02 ± 0.06
Lee^28^	0.90 ± 0.08	0.88 ± 0.10	0.002 ± 0.003	0.14 ± 0.19
Wan^7^	0.93 ± 0.04	0.93 ± 0.05	0.001 ± 0.002	0.11 ± 0.13
Wang^23^	0.90 ± NA	0.93 ± NA	NA	0.068 ± NA
Ours	0.97 ± 0.02	0.96 ± 0.02	0.0007 ± 0.0006	0.006 ± 0.033

The values are in the format of $\mu \pm \sigma$.

**Table 4:** Comparison of segmentation performance on ISBI 2015 testing dataset using DSC, TPRp, FPRp, FNRo (DSC threshold = 0.7). The values are in the format of $\mu \pm \sigma$.  ^∗^ The test dataset consists of 210 images of size $224\times 224$ extracted from real EDF images of size $1024\times 1024$ on ISBI2015.

Methods	DSC	TPRp	FPRp	FNRo
Tareef^9^	0.85 ± NA	0.94 ± NA	0.002 ± NA	0.34 ± NA
Song^5^	0.89 ± NA	0.92 ± NA	0.002 ± NA	0.26 ± NA
Lee^28^	0.88 ± 0.09	0.88 ± 0.12	0.001 ± 0.001	0.43 ± 0.17
Phoulday^20^	0.87±NA	0.88 ± NA	NA	0.21 ± NA
Wan*^,^^7^	0.92 ± 0.05	0.91 ± 0.05	0.001 ± 0.003	0.24 ± 0.19
Wang^23^	0.88 ± NA	0.85 ± NA	NA	0.32 ± NA
Ours	0.88 ± 0.02	0.92 ± 0.04	0.001 ± 0.0002	0.22 ± 0.12

**Table 5:** Comparison of segmentation performance on the two datasets using DSC, TPRp, FPRp, FNRo (DSC threshold = 0.8). The values are in the format of $\mu \pm \sigma$.  ^∗^ The test dataset consists of 210 images of size $224\times 224$ extracted from real EDF images of size $1024\times 1024$ on ISBI2015.

Dataset	Methods	DSC	TPRp	FPRp	FNRo
ISBI2014	Tareef^9^	0.92 ± NA	0.95 ± NA	0.003 ± NA	0.20 ± NA
	Lu^25^	0.96 ± 0.03	0.96 ± 0.04	0.002 ± 0.004	0.11 ± 0.15
	Wan^7^	0.94 ± 0.04	0.92 ± 0.04	0.002 ± 0.002	0.15 ± 0.17
	Ours	0.97 ± 0.02	0.97 ± 0.03	0.0007 ± 0.0005	0.013 ± 0.05
ISBI2015	Tareef^9^	0.89 ± NA	0.97 ± NA	0.002 ± NA	0.59 ± NA
	Wan*^[,7^	0.91 ± 0.06	0.90 ± 0.05	0.001 ± 0.002	0.28 ± 0.24
	Ours	0.92 ± 0.02	0.93 ± 0.03	0.0008 ± 0.0001	0.38 ± 0.14

**Figure 5:**
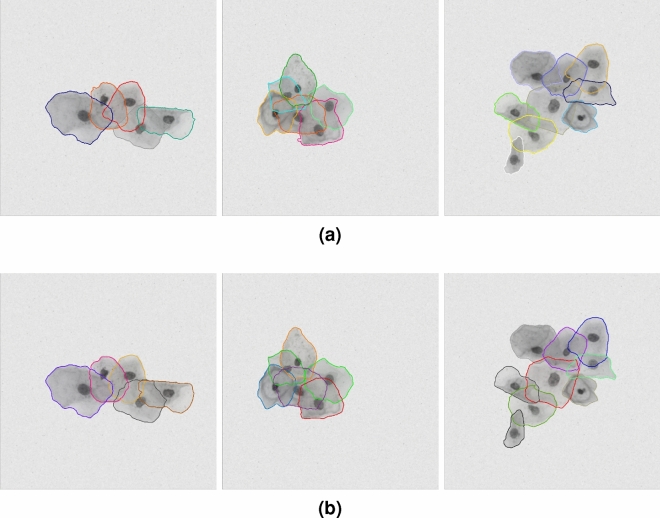
Segmentation results of typical images from ISBI2014: (**a**) ground-truth cytoplasm segmentation; (**b**) predicted cytoplasm segmentation by our model.

**Figure 6:**
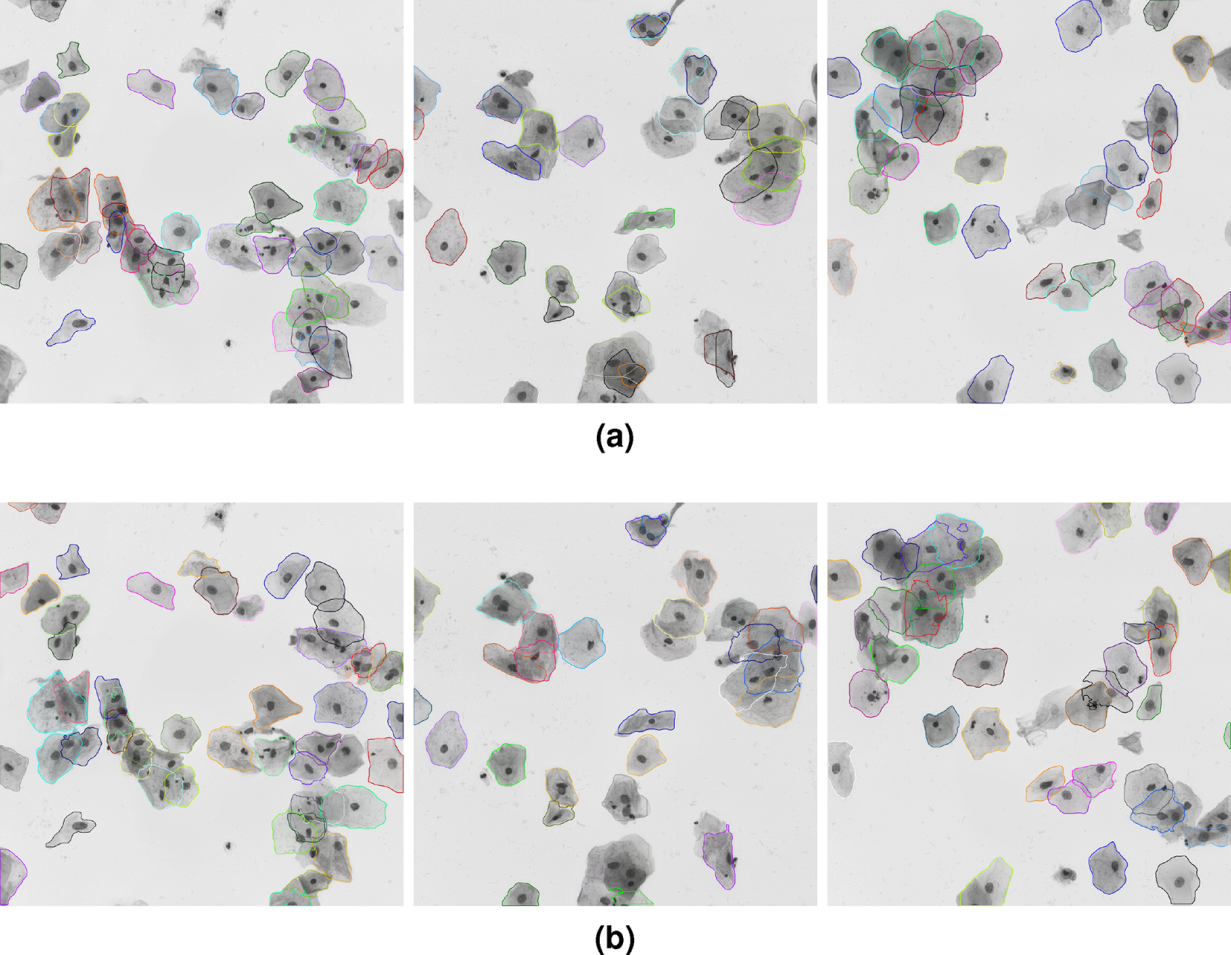
Segmentation results of typical images from ISBI2015: (**a**) ground-truth cytoplasm segmentation; (**b**) predicted cytoplasm segmentation by our model.

Figures 5 and 6 show the predicted cytoplasm segmentation results and the ground-truth annotations for some typical images from both datasets. As can be seen from those qualitative results, the cell masks in synthetic images are more accurate than the masks in real EDF images; Also, the masks of cells with low overlapping rate are much more precise than the masks of cells in dense cell clumps.

To further demonstrate the effectiveness of our method, a typical image from ISBI2014 dataset is used to show the cell segmentation results visually. The images from Ushizima^17^, Nosrati^29^, Lu^25^ and Tareef^9^ are used in this paper for comparison.

**Figure 7:**
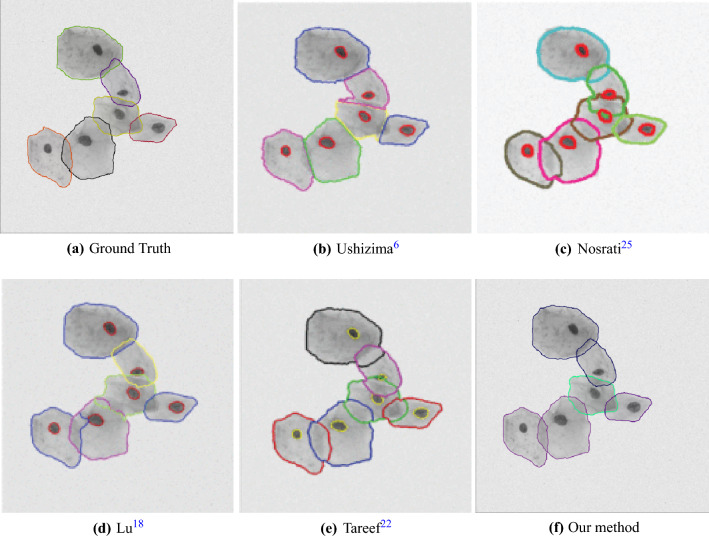
Visual comparison of cytoplasm segmentation results between typical methods. (**a**) Ground-truth cytoplasm segmentation. Images in (**b**–**e**) are provided by Tareef^9^ (It should be noticed that those four images also contain the segmentation of cell nuclei). (**f**) Predicted cytoplasm segmentation by our method.

Figure 7 shows that the predicted edges of cell cytoplasm are more smooth and accurate than that of state-of-the-art method.

**Figure 8:**
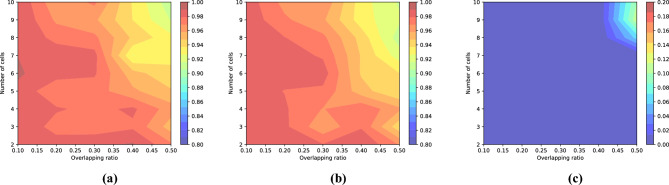
Visualization of segmenting results with different cell numbers and overlapping rates: (**a**) evaluation by DSC; (**b**) evaluation by TPRp; (**c**) evaluation by FNRo.

Furthermore, the dataset published by the ISBI2014 challenge contains not only the cytoplasm annotations, but also the number and average overlap rate of cells in each image. The number of cells ranges from 2 to 10 and the average overlap rates range from 0 to 0.5. Based on those facts, the performance of our segmentation method with various cell numbers and overlapping rates can be evaluated. Figure 8 clearly shows the change trend of segmentation performance with the number of cells and the overlap rates.

In summary, our model performs very well on cervical cytology images with relatively low overlap rate. For real EDF images with high overlap rate, the total performance of the proposed method is above the average level. Whereas our model might fail to precisely detect all the edge pixels of cytoplasm in the most dense cell clumps.

### Ablation experiments

We conduct a number of ablation experiments to analyze our model. The experiments were carried out from two aspects. Two important hyperparameters are the number (defined as $\alpha$) of FC layers in the point head and the number (defined as $\beta$) of points selected for calculating the uncertainties. Specifically, three different numbers (2, 3 and 4) for the FC layers in point head and two numbers (196 and 784) for selected points are used in the ablation experiments. The results of ablation experiments are listed in Table 6.

**Table 6:** The performance with different hyperparameters: $\alpha =$ the number of FC layers in point head and $\beta =$ the number of points selected for computing the uncertainties. The metrics are calculated with DSC threshold = 0.7.

	DSC	TPRp	FPRp	FNRo
ISBI2014				
$\alpha =3,~\beta =14\times 14$	0.967 ± 0.024	0.965 ± 0.026	0.0007 ± 0.0006	0.006 ± 0.033
$\alpha =4,~\beta =14\times 14$	0.966 ± 0.025	0.964 ± 0.026	0.0008 ± 0.0006	0.005 ± 0.023
$\alpha =3,~\beta =28\times 28$	0.966 ± 0.024	0.963 ± 0.026	0.0007 ± 0.0006	0.009 ± 0.036
$\alpha =4,~\beta =28\times 28$	0.966 ± 0.024	0.962 ± 0.027	0.0007 ± 0.0005	0.006 ± 0.031
$\alpha =2,~\beta =14\times 14$	0.965 ± 0.026	0.962 ± 0.027	0.0008 ± 0.0006	0.004 ± 0.021
Without point head	0.93 ± 0.029	0.90 ± 0.048	0.001 ± 0.0007	0.022 ± 0.066
ISBI2015				
$\alpha =3,~\beta =14\times 14$	0.883 ± 0.021	0.903 ± 0.041	0.001 ± 0.0002	0.218 ± 0.123
$\alpha =4,~\beta =14\times 14$	0.878 ± 0.022	0.898 ± 0.037	0.001 ± 0.0002	0.206 ± 0.112
$\alpha =3,~\beta =28\times 28$	0.877 ± 0.023	0.901 ± 0.039	0.001 ± 0.0002	0.209 ± 0.098
$\alpha =4,~\beta =28\times 28$	0.882 ± 0.022	0.887 ± 0.041	0.0009 ± 0.0002	0.219 ± 0.111
$\alpha =2,~\beta =14\times 14$	0.886 ± 0.020	0.902 ± 0.032	0.001 ± 0.0003	0.248 ± 0.126
Without point head	0.88 ± 0.022	0.91 ± 0.051	0.001 ± 0.0002	0.277 ± 0.093

As can be seen from Table 6, different combinations of hyperparameters $\alpha$ and $\beta$ do not affect the segmentation performance much. Quantitatively, the metrics of DSC and TPRp fluctuate within $1\%$ in most cases. But the point head improves the performance of DSC, TRPp and FNRo by about $3\%$, $6\%$ and $1.6\%$ respectively on dataset ISBI2014, compared to the original Mask RCNN. The point head improves the metric of FNRo from $27.7\%$ to $21.8\%$ on ISBI2015. However, the PointRend module dose not improve the performance of other metrics as much on ISBI2015 as on ISBI2014.

On the other hand, the model was trained for more than 500 epochs, but the total loss and other evaluation metrics got negligible performance improvement. In a word, longer training delivers similar results.

## Discussion

Analyzing every cervical cell in pathological images, obtained from the Pap smear test, is a very important task for early diagnosis of cervical cancer. The shapes, diameters and volumes of cells are crucial features for determining the degree of pre-cancerous lesions. Cervical cell segmentation could present more detailed information than those features.

The cervical cytology images from ISBI2015 not only have much more cells but also have higher overlap rates than the images from ISBI2014. Figure 1 shows some typical cervical images with their cytoplasm annotations. As for the dataset from ISIB2014, Table 3 presents the cell segmentation performance of seven methods (including ours). Our method outperforms state-of-the-art by moderate margin. In detail, the metric DSC and TPRp of our model is 0.97 and 0.96, respectively. Compared with the best results of selected methods, DSC and TPRp obtain improvement by 3% and 1%, respectively. The metric FNRo is 0.006, that is much lower than that of selected methods. Small value of FNRo means that almost all cells are successfully detected by our model. Figure 8 shows the overall segmentation performance by visualization. As for the dataset from ISBI2015, Table 4 gives the detailed comparison results. The performance of our method is slightly above the average level. None of the methods has achieved superior performance in four metric terms. This situation may be caused by the following facts. (1) Cells in each image overlap at a very high rate and their boundaries are very blur, to make accurately segmenting all cells extremely hard; (2) It’s a very tedious and time-consuming task to precisely annotate the boundary of every single cell in cervical cytology images. Therefore cervical images with high quality annotations are rare and precious. The dataset from ISBI2015 contains only nine images of this kind. Qualitatively, Figs. 5 and 6 show several cervical image examples with ground-truth annotations and predicted cytoplasm segmentation. The boundaries of cells having low overlap rates are much more precise than the boundaries of cells in clumps. Most of the cells that are not detected by our model also lie in the cell clumps. Figure 7 shows a visual comparison of cytoplasm segmentation results on a specific image from ISBI2014 between typical methods. During the training process, one iteration takes about 50 s. 8 h are needed to run 500 epochs. While evaluating, it takes about 0.05 s to obtain the segmentation results on one cervical cytology image. It should be noticed that the time efficiency depends largely on the hardware GPU. In summary, the qualitative and quantitative evaluations demonstrate the efficiency of our proposed methodology for overlapping cell segmentation in cervical images.

However, Tables 3 and 4 show that our model does not outperform existing methods as significantly on dataset ISBI2015 as on dataset ISBI2014. As can be seen from Figs. 5 and 6, the edges of cytoplasm in dense cell clumps in a image from ISBI2015 are much more blurred than that in a image from ISBI2014. In some cases, the edges are indistinguishable even for human experts. The PointRend module uses a small multilayer perceptron to refine the candidate edge pixels based on their certainties. In this way, if a pixel in dense cell clumps is not predicted as a candidate edge pixel by the backbone network, it would not be refined by the PointRend module. It’s worth mentioning that a pixel may lie on the edge of one cytoplasm and be inside the other cytoplasm at the same time. Therefore, the point head in our model dose not contribute much while segmenting cytoplasm from highly overlapped cell clumps. In short, a disadvantage of our method is that it might fail to effectively extract features of the boundary pixels which lie in the most blurred regions.

To overcome the disadvantage of the proposed method, strengthening the dataset and improving the structure of network theoretically are two possible ways. Firstly, the training dataset only contains 863 images, in which there are 8 real EDF images. In spite of data augmentation, the number of original images is a little small for convolutional neural network. Thus strengthening the original dataset is an essential way to improve the model. Zhao et al.^30^ proposed a method by using a point annotation, which was much easier than completely manual annotations. This weakly supervised method could be used with manual annotation, to alleviate the stresses of human experts. Secondly, traditional instance segmentation models extract local features by handcrafted anchor boxes, which may be not efficient for detecting the edge pixels from highly blurred regions. Long-distance features are also neglected. Therefore both local and long-distance features should be taken into consideration, in order to improve the performance of segmenting cells from dense clumps. The mechanism of self-attention incorporates local and global features, to make it a candidate method for improving the proposed model.

## Conclusion and future works

In this paper, we present a novel convolutional neural network based on PointRend to address the challenging task of segmenting every single cell in cervical cytology images. The main idea is that fine grained features and coarse features are extracted from different feature maps to fine-tune the boundary pixels of low certainty. The experiment results on publicly available datasets show that our approach outperforms state-of-the-art methods by moderate margin on dataset from ISIB2014 and is slight better than the average level on dataset from ISBI2015. Concretely, our model outperforms state-of-the-art method by 3% on DSC, 1% on TPRp and 1.4% on FNRo respectively on dataset ISBI2014, meanwhile the metric of FNRo is much lower than other methods. However, the averaged metrics DSC, TRPp, FNRo and FPRp on dataset ISBI2015 are 0.88, 0.89, 0.3 and 0.0015, respectively. Our model obtains results a little better than the averaged values on four metrics. Our future work will focus on two possible aspects to improve the performance of segmentation. One is that weakly supervised learning would be introduced and tested for cell segmentation. The other is that deep learning module that extract global features such as transformer would be added into our model.

## Data Availability

The two datasets analysed during the current study were presented and made publicly available during the first and second Overlapping Cervical Image Segmentation Challenges in 2014 (https://cs.adelaide.edu.au/~carneiro/isbi14_challenge/dataset.html) and 2015 (https://cs.adelaide.edu.au/~carneiro/isbi15_challenge/dataset.html).
